# Burden of non-communicable disease studies in Europe: a systematic review of data sources and methodological choices

**DOI:** 10.1093/eurpub/ckab218

**Published:** 2022-01-07

**Authors:** Periklis Charalampous, Vanessa Gorasso, Dietrich Plass, Sara M Pires, Elena von der Lippe, Alibek Mereke, Jane Idavain, Katarzyna Kissimova-Skarbek, Joana Nazaré Morgado, Che Henry Ngwa, Isabel Noguer, Alicia Padron-Monedero, María José Santi-Cano, Rodrigo Sarmiento, Brecht Devleesschauwer, Juanita A Haagsma, The COST Action CA18218 Participants, Balázs Ádám, Ala’a Alkerwi, Boris Bikbov, Anette Kocbach Bølling, Susanne Breitner, Sarah Cuschieri, Christina C Dahm, Terje Andreas Eikemo, Florian Fischer, Alberto Freitas, Juan Manuel García-González, Federica Gazzelloni, Mika Gissler, Brigita Hengl, Paul Hynds, Gaetano Isola, Lea S Jakobsen, Zubair Kabir, Ann Kristin Knudsen, Naime Meriç Konar, Carina Ladeira, Aaron Liew, Marjeta Majer, Enkeleint A Mechili, Vildan Mevsim, Milena Santric Milicevic, Louise Mitchell, Lorenzo Monasta, Stefania Mondello, Evangelia Nena, Edmond S W Ng, Vikram Niranjan, Rónán O'Caoimh, Mark Ryan O’Donovan, Alberto Ortiz, Elena Pallari, Panagiotis Petrou, Miguel Reina Ortiz, Silvia Riva, Hanène Samouda, João V Santos, Cornelia Melinda Adi Santoso, Tugce Schmitt, Dimitrios Skempes, Ana Catarina Sousa, Aleksandar Stevanovic, Gerhard Sulo Natasa Terzic, Zorica Terzic-Supic, Jovana Todorovic, Fimka Tozija, Brigid Unim, Lisa Van Wilder, Orsolya Varga, Francesco S Violante, Grant M A Wyper

**Affiliations:** 1 Department of Public Health, Erasmus MC, University Medical Center, Rotterdam, The Netherlands; 2 Department of Public Health and Primary Care, Ghent University, Ghent, Belgium; 3 Department of Epidemiology and Public Health, Sciensano, Brussels, Belgium; 4 Department for Exposure Assessment and Environmental Health Indicators, German Environment Agency, Berlin, Germany; 5 National Food Institute, Technical University of Denmark, Lyngby, Denmark; 6 Department of Epidemiology and Health Monitoring, Robert Koch Institute, Berlin, Germany; 7 Al-Farabi Kazakh National University, Almaty, Kazakhstan; 8 National Institute for Health Development, Tallinn, Estonia; 9 Department of Health Economics and Social Security, Jagiellonian University Medical College, Krakow, Poland; 10 Environmental Health and Nutrition Laboratory, Faculty of Medicine, University of Lisbon, Lisbon, Portugal; 11 School of Public Health and Community Medicine, Sahlgrenska Academy, University of Gothenburg, Gothenburg, Sweden; 12 Carlos III Institute of Health, National School of Public Health, Madrid, Spain; 13 Research Group on Nutrition: Molecular, pathophysiological and social issues, Biomedical Research and Innovation Institute of Cádiz (INiBICA), University of Cádiz, Cádiz, Spain; 14 Medicine School, University of Applied and Environmental Sciences, Bogota, Colombia; 15 Department of Translational Physiology, Infectiology and Public Health, Ghent University, Merelbeke, Belgium

## Abstract

**Background:**

Assessment of disability-adjusted life years (DALYs) resulting from non-communicable diseases (NCDs) requires specific calculation methods and input data. The aims of this study were to (i) identify existing NCD burden of disease (BoD) activities in Europe; (ii) collate information on data sources for mortality and morbidity; and (iii) provide an overview of NCD-specific methods for calculating NCD DALYs.

**Methods:**

NCD BoD studies were systematically searched in international electronic literature databases and in grey literature. We included all BoD studies that used the DALY metric to quantify the health impact of one or more NCDs in countries belonging to the European Region.

**Results:**

A total of 163 BoD studies were retained: 96 (59%) were single-country or sub-national studies and 67 (41%) considered more than one country. Of the single-country studies, 29 (30%) consisted of secondary analyses using existing Global Burden of Disease (GBD) results. Mortality data were mainly derived (49%) from vital statistics. Morbidity data were frequently (40%) drawn from routine administrative and survey datasets, including disease registries and hospital discharge databases. The majority (60%) of national BoD studies reported mortality corrections. Multimorbidity adjustments were performed in 18% of national BoD studies.

**Conclusion:**

The number of national NCD BoD assessments across Europe increased over time, driven by an increase in BoD studies that consisted of secondary data analysis of GBD study findings. Ambiguity in reporting the use of NCD-specific BoD methods underlines the need for reporting guidelines of BoD studies to enhance the transparency of NCD BoD estimates across Europe.

## Introduction

In the early 1990s, the World Bank published the first Global Burden of Disease and Injury (GBD) study, introducing the Disability-Adjusted Life Years (DALYs) as a key metric for assessing the burden of disease (BoD) in populations.^1–3^ One important feature of the DALY metric is that it aggregates populations’ health losses into a single figure summarizing mortality, measured by Years of Life Lost (YLLs), and morbidity, measured by Years Lived with Disability (YLDs).^3^^,^^4^ The DALY is the key element of the BoD approach: a framework for integrating all available information on fatal and non-fatal health outcomes to provide an overview of the causes of health loss. Over the years, the BoD approach has informed public health policy, since it allows for comparison of disease impact on population health across different health conditions and populations as well as over time.^3^^,^^4^

The GBD 2019 study estimated that non-communicable diseases (NCDs) accounted for almost 90% of deaths and more than 80% of DALYs in the European Region.^5^ NCDs comprise diseases that are heterogeneous in terms of case-fatality rates and severity of non-fatal health outcomes, and many data sources are needed to estimate DALYs resulting from NCDs. Moreover, the presence of multimorbidity (i.e. the co-occurrence of multiple morbid conditions in the same individual) is common in persons with NCDs, especially with increasing age.^6^ Assessing simultaneous conditions as separate entities leads to an overestimation of the disease burden due to double counting.^6^^,^^7^ Another major methodological choice for calculating NCD YLDs is whether to use an incidence or prevalence approach.^8^ For YLDs, the difference between these perspectives has to do mainly with the period to which morbidity is assigned. Incidence-based YLD estimates are calculated based on newly diagnosed cases and the duration of a condition to capture the future stream of disease burden.^3^ Prevalence-based YLD estimates quantify current disease burden by taking the prevalent cases at a specified point in time.^3^ Hence, for assessing disease burden due to NCDs, the prevalence-based approach might have some advantages over the incidence-based approach; first, because the incidence of some NCDs is not precisely measurable; second, because the number of current prevalent cases is the result of the new and pre-existing cases; and third, the information on duration is not easily available and also varies widely.^9^^,^^10^ To estimate YLLs, the incidence-based approach is universally applied due to the nature of death event.^3^^,^^8^

A mapping activity examining BoD studies conducted by O’Donovan *et al*. identified a total of 198 studies published between 1997 and mid-2016 for countries in the World Health Organization (WHO) European Region, including over twenty full national or sub-national BoD assessments. This study has already indicated that methodological choices for assessing disease burden are not harmonized.^11^ However, this review did not elaborate on NCD-specific data sources nor methodological choices, such as multimorbidity adjustments. Insight into these aspects may shed light on methods and data sources that have been used over the years and also help to critically discuss the comparability of the results of NCD-related BoD studies across Europe.

In a first step, we identify existing NCD BoD activities in Europe. In a second step, we collate the information on data sources for mortality and morbidity; and in a third step, we provide an overview of the NCD-specific methodological choices for calculating BoD.

The following key questions were addressed:

- How many NCD BoD assessments have been performed across Europe, and in which European countries?
- Which mortality and morbidity data sources have been used as input data for NCD BoD assessments?
- Which NCD-specific methodological choices have been used in these BoD studies?

## Methods

This systematic literature review was conducted following the guidance produced by the Centre for Reviews and Dissemination (CRD) as well as the Preferred Reporting Items for Systematic Reviews and Meta-analyses (PRISMA) Statement.^12^^,^^13^ The study protocol has been registered on PROSPERO, number CRD42020177477 (available at: https://www.crd.york.ac.uk/PROSPERO/). This review is part of a series of systematic reviews launched by the *burden-eu* network,^14^ aiming to explore and harmonize the BoD methodological choices used in BoD studies across Europe. The *burden-eu* network actively works to establish a quality assessment framework for BoD studies.

### Data sources and search strategy

The databases Medline, Embase, Cochrane and Web of Science were systematically searched, using search terms covering YLL, YLD and/or DALY calculations based on Group I: communicable, maternal, neonatal, and nutritional diseases; Group II: NCDs; and Group III: Injuries. The search strategy was developed by an experienced librarian from the Erasmus MC University, in April 2020. Search strings, including the Boolean operators used, are provided in Supplementary file (p. 4). A grey literature search was also carried out including (i) grey literature databases (i.e. OpenGrey, OAIster, CABDirect, and WHO) and (ii) targeted websites of public health agencies (Supplementary file, p. 7). Additionally, *burden-eu* members were contacted to provide any further eligible publications that might have been missed by the search strategy. A hand search within the reference lists was also performed to identify eligible BoD studies, review studies and reports not originally flagged by the search strategy.

### Eligibility criteria

Peer-reviewed articles and grey literature published between January 1990 and April 2020 were included. We did not consider BoD studies that were published before 1990, since the DALY concept was introduced in the early 1990s. Studies that quantified YLLs, YLDs, and/or DALYs covering overall disease burden (i.e. Group I, Group II, and Group III) or one or multiple NCD-specific health outcomes were included (Supplementary file, p. 3). Thus, BoD studies that quantified disease burden only due to Group I and/or Group III and/or risk factors were excluded from our analysis. Only BoD studies conducted within the European countries as defined by the GBD area “European Region” (Supplementary file, p. 2) were considered. No language restrictions were applied. Publications with missing detailed methodological information, such as conference proceedings, abstracts, editorials, letters to editors and general correspondence were excluded.

### Data screening, selection and extraction

After generating a database of unique titles, titles (first step), abstracts (second step) and, if needed, full-texts (third step) were screened for eligibility. The screening process was conducted independently by two researchers (PC and VG) using EndNote X9 software. Disagreement about eligibility was resolved by discussion and if needed through the study supervisor (JH). Finally, a database of all retrieved publications that met the eligibility criteria listed above was compiled. For each of the eligible papers, information was extracted using a data extraction grid. The information collected was related to the following items: study information and characteristics, data sources for mortality, data sources for morbidity, data adjustments, internal consistency, methodological perspectives used to calculate YLLs and/or YLDs, and multimorbidity adjustment. Definitions of the extraction items are provided in the Supplementary file (p. 8). Data extraction was performed by PC and discussed with VG, and for the non-English papers by the *burden-eu* native speakers.

### Synthesis of study results

Eligible BoD studies are primarily classified by their geographic coverage (i.e. multi-country or single-country) and/or by the type of analysis (i.e. analysis of BoD by calculating own DALYs, YLDs or YLLs or secondary or systematic analysis using existing GBD estimates). Two secondary classifications of the identified BoD studies are also presented. First, all BoD studies are categorized by ‘year of publication’ and, second, by the ‘cause of ill-health category’ (i.e. overall BoD assessment that quantified BoD due to Group I, Group II and Group III or NCD-specific assessment that quantified BoD resulting from 12 NCD-specific categories).

## Results

### Number of burden of disease studies identified

**Figure 1:**
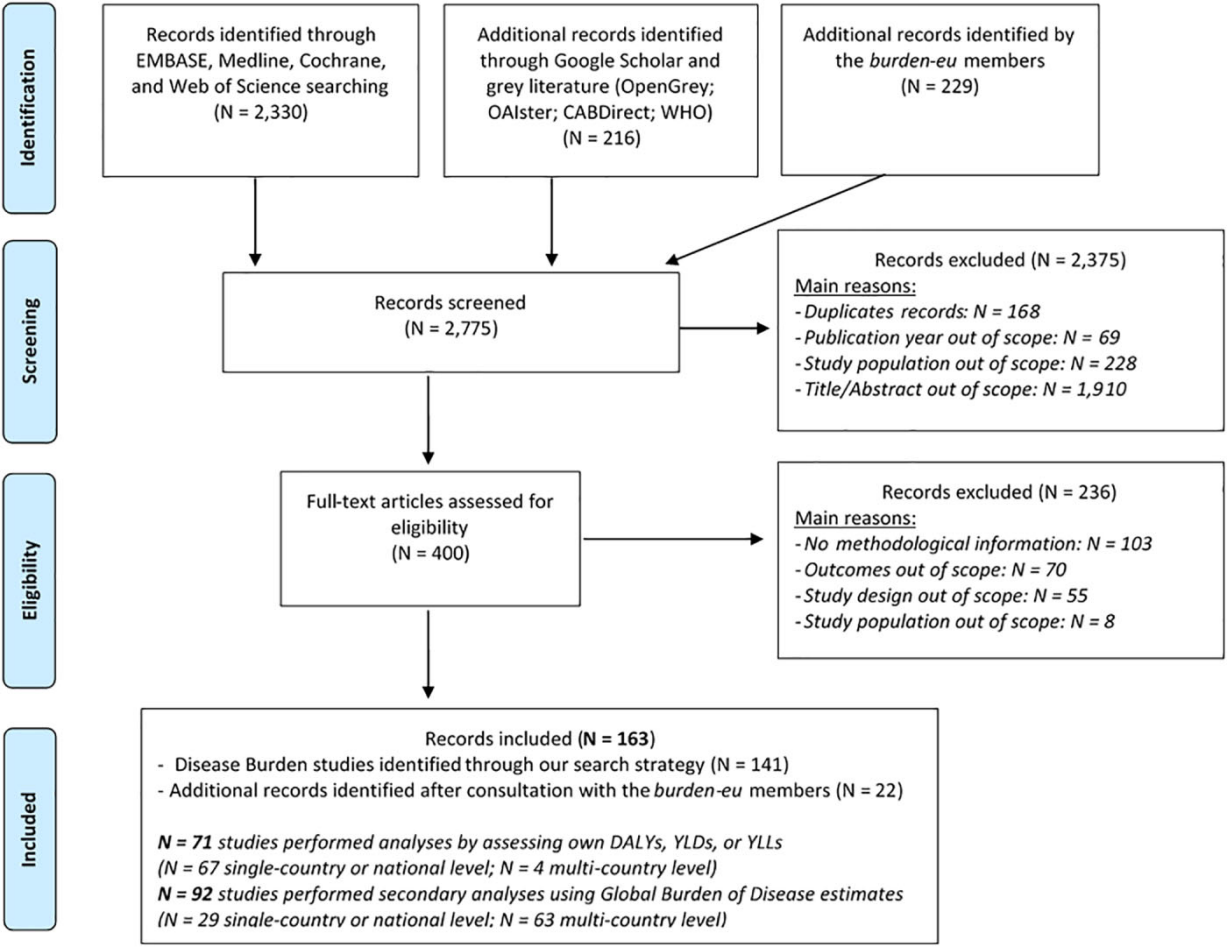
Flowchart of the search of existing burden of disease studies in the ‘Global Burden of Disease’ European region countries

Figure 1 shows the flowchart for the performed search, the main reasons for exclusion, and the total number of eligible studies. In total, we retained and evaluated 163 BoD studies; identified 141 records through our search strategy; and a further 22 after consultation with *burden-eu* members.

### Number of burden of disease studies by ‘country type’

**Figure 2:**
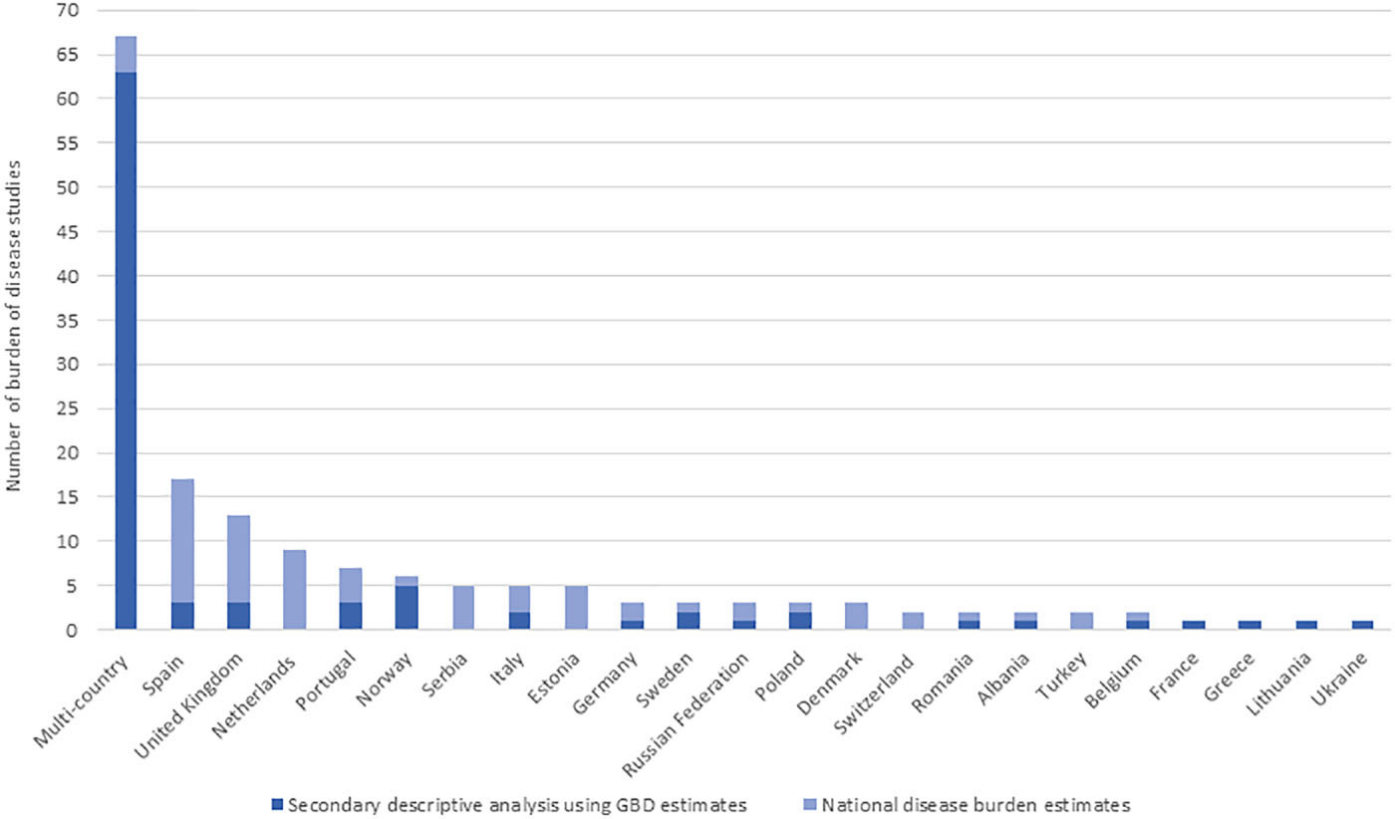
Number of existing burden of disease assessments per European country. *Please note that the number of the identified studies refers to BoD assessments performed between the January 1990 and April 2020 period

Of these 163 studies, 96 (59%) were single-country BoD studies, and 67 (41%) were multi-country BoD studies. Of the single-country studies, 67 (70%) consisted of analysis of disease burden estimates by calculating own DALYs, YLDs or YLLs, whereas 29 (30%) consisted of secondary analyses using GBD results. The single-country BoD studies were performed in 22 European countries. The largest number of the single-country BoD assessments were observed in Spain (*N* = 17), the United Kingdom including England, Wales, Scotland and Northern Ireland (*N* = 13), and the Netherlands (*N* = 9). Sixty-three (94%) multi-country BoD studies used GBD results. Figure 2 illustrates the number of existing BoD assessments per European country.

### Number of burden of disease studies by ‘year of publication’

**Figure 3:**
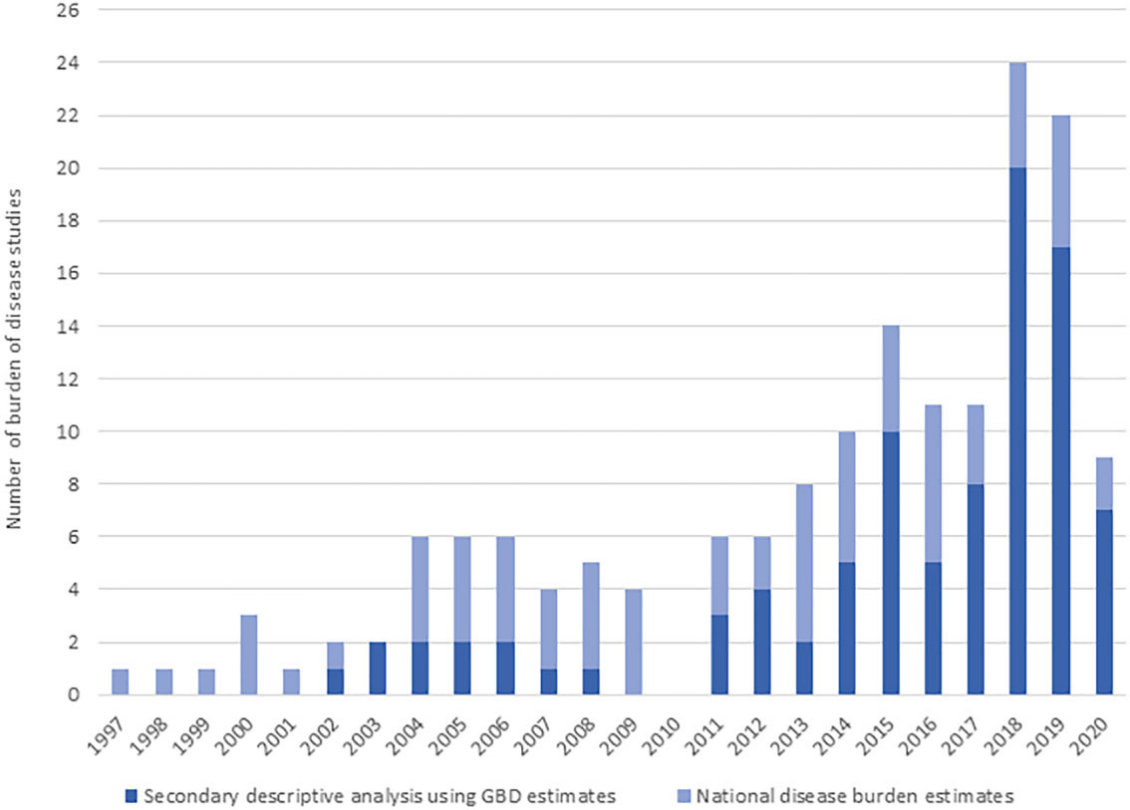
Total number of existing burden of disease assessments by year of publication. *Please note that the number of the identified studies refers to BoD assessments performed between January 1990 and April 2020. **There were no studies published in 2010 that met our eligibility criteria

Figure 3 shows the total number of existing BoD assessments by year of publication. Between 1997 and 2010, the number of studies that consisted of analysis of BoD estimates by calculating own DALYs, YLDs or YLLs was consistently higher compared to the number of studies carrying out secondary or systematic analyses using GBD estimates. After 2010, the number of studies performing secondary or systematic analyses based on GBD results steadily increased. The top-two years with a higher number of BoD studies using GBD results were 2018 (*N* = 20) and 2019 (*N* = 17), while the number of the conducted national or sub-national BoD studies was four and five, respectively.

### Number of burden of disease studies by ‘cause of ill-health outcomes’

Out of a total 163 BoD studies, 74 presented an overall BoD assessment (i.e. Group I, Group II, and Group III), and 89 presented an NCD-specific assessment. More than half of the NCD-specific BoD studies (*N* = 49; 55%) assessed the burden of multiple NCDs, while the remaining 40 (45%) covered a single NCD. Briefly, the NCD disease groups covered in the NCD-specific assessments were: neoplasms (*N* = 25); neurological disorders (*N* = 14); cardiovascular diseases (*N* = 13); mental disorders and substance use disorders (*N* = 11); other NCDs (*N* = 8); musculoskeletal disorders (*N* = 6); digestive diseases (*N* = 4); chronic respiratory diseases (*N* = 3); diabetes and kidney diseases (*N* = 3); and skin and subcutaneous diseases (*N* = 2).

### Data input sources mortality and morbidity

**Figure 4:**
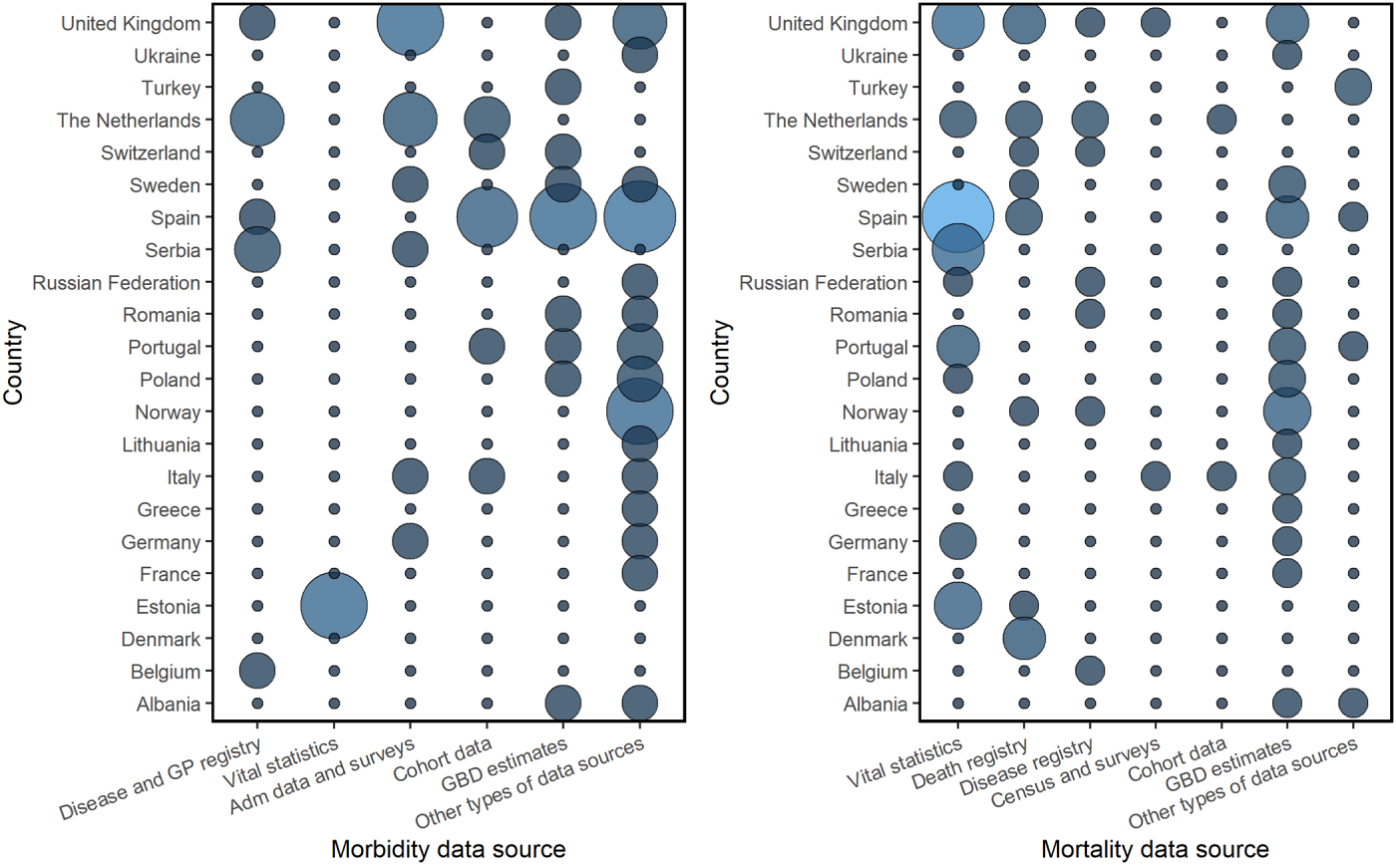
Data input sources for morbidity (left) and mortality (right) in the single-country burden of disease studies (*N* = 96) performed in 22 European countries. *Adm: Administrative data; GBD: Global Burden of Disease; GP: General Practitioner. **Please note that the size and the colour of each circle correspond to the amount of burden of disease studies for each European country in each data source for morbidity (left) and mortality (right). The bigger (and lighter) the circle, the higher the number of BoD studies

Various types of data sources were used to collect input data for BoD studies. Figure 4 shows the data input sources for mortality and morbidity in single-country BoD studies (*N* = 96) performed in 22 European countries. Twenty-nine single-country BoD studies obtained mortality and/or morbidity estimates from the GBD study. Data sources for fatal and non-fatal data in national or sub-national BoD studies (*N* = 67) differ markedly.

The vast majority of the national or sub-national BoD studies derived mortality data directly from vital statistics, using the WHO International Statistical Classification of Diseases and Related Health Problems. Disease registry data and bureau of population censuses and surveys were also used to assess the fatal BoD in many national BoD studies (Belgium, the Netherlands, the United Kingdom, Italy, Russian Federation, etc.). Verbal autopsy data were used in the Turkish BoD study. Few BoD studies (6%) obtained fatal data from cohort studies (e.g. the Netherlands, Italy). The remaining national or sub-national BoD studies used multiple mortality data input sources.

The disease morbidity estimates were also drawn from various data input sources. Routine administrative and survey datasets, including disease registries, hospital discharges, and general practitioner (GP) registration systems, were mostly used in national or sub-national BoD studies (e.g. Scotland, the Netherlands, Spain, Sweden, Belgium, etc.). The Estonian BoD study derived YLD estimates from the Estonian Health Insurance Fund database. The remaining national or sub-national BoD studies used a variety of additional data sources on incidence, prevalence, severity and duration of illnesses.

### Data adjustments mortality and morbidity

All studies that performed secondary or systematic analyses using GBD results (*N* = 92; 68% multi-country and 32% single-country) reported adjustments of mortality and/or morbidity data and of internally consistent estimates related to the methods used in the GBD study.

In total, 69 out of 71 studies (97%; 94% single-country and 6% multi-country) that presented BoD analyses by calculating own DALYs, YLDs or YLLs quantified disease burden using YLLs. Most of these studies (*N* = 41; 60%), reported corrections to the preliminary round of mortality estimates for the selected causes of death. Specifically, the reported adjustments were related to issues of (mis)-coding of causes of death. Ten (15%) single-country BoD studies (conducted in Turkey, Spain, and Germany) reported the development of an all-cause mortality envelope, in which the sex-redistributed and age-redistributed estimates of deaths were aggregated with the total number of deaths.

In total, 55 out of 71 studies (77%; 93% single-country and 7% multi-country) that calculated own BoD estimates included YLD calculations. Over half of these studies (*N* = 35; 64%) reported data adjustments of the estimates of incidence and prevalence. Eleven of these studies included the use of disease modelling software (DisMod) to prepare and ensure internal epidemiological consistency of incidence, prevalence, and disease duration estimates. Ten studies reported the use of GesMor software in performing BoD analyses. These studies were all conducted in Spain.

### Methodological choices

#### YLDs calculation: choice of an incidence-based or prevalence-based approach

In total, 86 out of 92 studies performed secondary or systematic analyses using GBD YLDs; 70 of these studies (81%) followed the prevalence-based approach, while 14 (17%) studies presented YLDs based on the incidence approach. The BoD studies quantifying YLDs based on the latter extracted non-fatal estimates from the original GBD 1990 study effort that calculated YLDs from incidence estimates.^1^ Two BoD studies (2%) did not report the approach used to calculate YLDs. On the other hand, 55 out of 71 studies that calculated own YLDs, 31 (56%) used the incidence-based approach and 20 (37%) used the prevalence-based approach. Four of these 55 BoD studies (7%) did not report the approach used to calculate YLDs.

#### Multimorbidity adjustment

Of the 92 studies that carried out secondary or systematic analyses using GBD results, about 20% used GBD estimates published before 2010 and multimorbidity corrections were not feasible. The remaining studies reported multimorbidity adjustments according to the micro-simulation framework, using multiplicative independence modelling. This framework, developed by GBD researchers, assumes no correlation between diseases and/or injuries by age and sex stratum.^9^ Of the 55 single-country and multi-country studies that calculated own non-fatal estimates, 10 (18%) studies reported multimorbidity adjustments for YLD calculations. These studies assessed YLDs based on the prevalence approach. Specifically, three countries (Germany, the Netherlands and Portugal) have adopted the GBD approach (i.e. multiplicative independence model) to adjust for multimorbidity. The Scottish BoD studies have developed methods to consider the influence of multimorbidity on the co-existing non-fatal outcomes; the Scottish BoD multimorbidity framework is also similar to the GBD approach. Three BoD studies reported multimorbidity adjustments, but not the methods used.

## Discussion

This systematic review provides a comprehensive overview of the number and types of NCD BoD activities performed in Europe until early-2020. Our aim was to collate information on data sources for NCD mortality and morbidity, and to summarize the NCD-specific methodological choices that were used in BoD assessments. In total, 163 BoD studies met our inclusion criteria. Over half of these studies were single-country or sub-national BoD studies undertaken in 22 European countries. The majority of single-country BoD assessments were performed in Western European countries, especially in Spain, the United Kingdom and the Netherlands. Furthermore, within Central Europe, the large percentage of Serbian BoD studies (42%) stand out and within Eastern Europe, the Estonian BoD studies (42%). On the other hand, for some European countries, such as Greece, France, Belarus and Cyprus, we identified a very low number or no BoD studies at all.

Of the single-country studies, the majority consisted of analyses of BoD estimates by calculating own DALYs, YLDs or YLLs, whereas approximately one in three consisted of secondary analyses using GBD results. However, over the last 23 years, we observed a shift in the proportion of studies that calculated own DALYs, YLDs or YLLs versus those that consisted of secondary analyses of GBD results, from 100% in the 1990s to 22% in early-2020. This indicates that more and more European countries and health agencies are using the GBD estimates to guide decisions concerning their populations’ health priorities. The benefit of using GBD study estimates lies in the comparativeness of its estimates—they are computed from a plethora of available data sources and adjusted using statistical modelling approaches—across regions and over time.^15^^,^^16^ This ensures BoD estimates for populations with sparse fatal or non-fatal data. Notwithstanding the above advantage, in some cases, national data sources, particularly morbidity data sources, are not considered in the GBD study. This could be, for instance, because the data source may be not accessible to the GBD researchers. Other explanations could be that national data sources are based on decentralized or fragmented data reporting systems and hence, the quality of the data is questioned or does not meet pre-specified criteria.

Our review reveals that the vast majority of the identified NCD-specific BoD activities focused on the health impact of neoplasms. Most of the neoplasm-related BoD studies were conducted in Western European countries. A possible explanation for this may be that neoplasms have been the leading cause of deaths and DALYs in Western Europe over the 1990–2019 period.^5^ Other explanations for this may be the availability of epidemiological data from population-based cancer registers,^17^ the introduction of neoplasm-related policy reforms or new treatments that are associated with improved survival outcomes for cancer patients, and therefore more BoD cancer research outputs to monitor changes over time were needed.^18^^,^^19^

Another finding of our review is that the vast majority of national or sub-national BoD studies undertaken in Europe obtained mortality data from vital statistic systems and death registrations, and morbidity data mainly from administrative data, and primary registration data such as GP registrations. Calculating BoD estimates require high-quality mortality and morbidity data. Regarding the mortality data, we found that some national BoD studies did not report the estimated completeness or the level of coverage for their (sub-)national registration systems. Reporting the level of completeness or the quality of cause-of-death data ensures sufficient information for decision-planning.^20^ Regarding the morbidity data input sources that have been used in national or sub-national BoD studies, we observed that few studies reported on data quality. Most of the identified national BoD studies assessed non-fatal estimates based on administrative data sources, while others obtained them from population-level health surveys. These data collection systems should have been evaluated for their level of ascertainment, relevance, and quality in order to obtain the most accurate morbidity estimates in national-level BoD studies.^7^ Hence, the development and use of key standardized guidelines for reporting the evaluation of mortality and morbidity data in national BoD studies is a critical priority. Such research efforts on the evaluation and use of more accessible, comparable and reliable BoD estimates may help decision-makers to draw national-level strategies.

Another noteworthy observation of this review is that very few national BoD studies have assessed the effects of multimorbidity with multimorbidity adjustment approach(es). We found that national studies that did not correct for multimorbidity quantified NCD YLDs based on the incidence perspective, where corrections for multimorbidity are more complicated, since the temporal effects of multi-morbid conditions need to be taken into account. However, by not adjusting for NCD multimorbidity, the morbidity component of DALYs might be overestimated. Hilderink *et al*. tested the impact of multimorbidity adjustments on YLDs for twenty-five NCD-specific conditions.^6^ When the NCD YLDs were calculated independently in combination with the multiplicative approach, they were 5% and 14% lower than when the additive and maximum limit method is applied, respectively.^6^ Therefore, analyzing the impact of multimorbidity of multiple NCDs on BoD estimates using other multimorbidity adjustment approaches than the independent multiplicative approach may result in an overestimation of the NCD YLDs. Similarly, in the GBD approach, the prevalence of multi-morbid conditions is estimated based on one independence assumptions.^5^^,^^9^ Some NCD BoD studies reported multimorbidity corrections, but not the methods used. This underlines the importance of using guidelines for performing NCD BoD studies as well as the need for reporting methodological guidelines of NCD BoD studies, since these will facilitate harmonization of methods as well as transparency and interpretation of BoD study results.

Our review has certain limitations. First, ongoing BoD studies or BoD studies that were performed but not documented in peer-reviewed articles or grey literature were not included, which could potentially have led to European NCD BoD studies being missed. Second, the search strategy was conducted in the English language, and non-English search terms have only been considered in the grey literature searches.

Our review showed that the number of national NCD BoD assessments across Europe increased over time and that this is due to the growth of single-country BoD studies that consist of secondary data analysis of GBD study findings. Reporting of NCD-specific BoD methods of studies that calculated own DALYs should be improved, underlining the need for reporting guidelines of BoD studies to enhance transparency, consistency and comparability of NCD BoD estimates at national-level and multi-level BoD activities across Europe.

## Supplementary data

Supplementary data are available at *EURPUB* online.

Key points

- A total of 163 BoD studies were identified: 96 were single-country or sub-national studies and 67 were multi-country studies.
- Of the single-country studies, the majority consisted of analyses of BoD estimates by calculating own DALYs, YLDs or YLLs, whereas approximately one in three consisted of secondary analyses using GBD results.
- The majority of single-country BoD studies obtained mortality data from vital statistics and morbidity data from routine administrative datasets.
- Multimorbidity adjustments were performed in very few (sub-) national BoD studies.
- Reporting of NCD-specific BoD methods of studies that calculated own DALYs should be improved, underlining the need for reporting guidelines of BoD studies.

## Supplementary Material

ckab218_Supplementary_Data
