# Supplementary material for: Burden of non-communicable disease studies in Europe: a systematic review of data sources and methodological choices
Source: Eur J Public Health. 2022 Jan 7;32(2):289–96. doi: 10.1093/eurpub/ckab218 (PMC8975530; doi:10.1093/eurpub/ckab218)
Supplement: ckab218_Supplementary_Data [file ckab218_supplementary_data.docx]

**Supplementary File**

**Burden of non-communicable disease studies in Europe: a systematic review of data sources and methodological choices**

Periklis Charalampous, Vanessa Gorasso, Dietrich Plass, Sara M. Pires, Elena von der Lippe, Mereke Alibek, Jane Idavain, Katarzyna Kissimova-Skarbek, Joana Nazaré Morgado, Che Henry Ngwa, Isabel Noguer, Alicia Padron-Monedero, María José Santi-Cano, Rodrigo Sarmiento, Brecht Devleesschauwer, Juanita A. Haagsma, and The COST Action CA18218 participants

Country selection ……………………………………………………….………………………………………………………….………………. 2

Overview of non-communicable diseases …………………………………………………………………….….…………………….. 3

Search strategy.……………………………………………………………………………………………………………………………………… 4

Grey literature search and websites of targeted national public health agencies ………………..…………………. 7

Definitions for the systematic review extraction form categories ………………………………….…………………….… 8

Reference list of included European burden of disease studies ……………………………………….……………………. 14

Data input sources for morbidity and mortality in single-country burden of disease studies ………………. 31

**Country Selection**

Burden of disease (BoD) studies from 45 European countries were searched. The selection of the European countries was made according to the Global Burden of Disease (GBD) categorisation. Box 1 shows the list of the GBD European countries*

**Box 1:** List of the GBD European countries

Albania, Andorra, Austria, Belarus, Belgium, Bosnia Herzegovina, Bulgaria, Croatia, Cyprus, Czech Republic, Denmark, Estonia, Finland, France, Germany, Greece, Hungary, Iceland, Ireland, Israel, Italy, Latvia, Lithuania, Luxembourg, Malta, Monaco, Montenegro, Netherlands, North Macedonia, Norway, Poland, Portugal, Republic of Moldova, Romania, Russian Federation, San Marino, Serbia, Slovakia, Slovenia, Spain, Sweden, Switzerland, Turkey, Ukraine, and United Kingdom

* Please note that:

(i) in this systematic review we decided to add also Turkey as a European country.

(ii) BoD studies undertaken at the constituent country or territory level were taken into account as well. For example, an eligible BoD study conducted in Greenland is categorised as a Danish BoD study. Also, eligible BoD studies covering England and/or Wales and/or Scotland and/or Northern Ireland are categorised as a United Kingdom BoD study.

**Overview of non-communicable diseases**

Non-communicable disease (NCDs) selection was made according to the Global Burden of Disease (GBD) 2019 cause hierarchy list*. Box 2 shows the NCDs considered in the NCD-specific BoD studies.

| **Box 2:** List of NCD-specific health outcomes |
| --- |
| Neoplasms |
| Cardiovascular diseases |
| Chronic respiratory diseases |
| Digestive diseases |
| Neurological disorders |
| Mental disorders |
| Substance use disorders |
| Diabetes and kidney diseases |
| Skin and subcutaneous diseases |
| Sense organ diseases |
| Musculoskeletal disorders |
| Other non-communicable diseases (i.e., Oral disorders, congenital birth defects, urinary diseases and male infertility, gynaecological diseases, endocrine, metabolic, blood, and immune disorders, hemoglobinopathies and haemolytic anemias, and sudden infant death syndrome) |
| * Global Burden of Disease Collaborative Network. Global Burden of Disease Study 2019 (GBD 2019) Cause, REI, and Location Hierarchies. Seattle, United States of America: Institute for Health Metrics and Evaluation (IHME), 2020  <https://doi.org/10.6069/Q0YC-CR46> |

**Search strategy**

**Embase**

('disability-adjusted life year'/de OR (DALY OR DALYs OR ((disabil*) NEAR/4 (adjust*) NEAR/4 (life*) NEAR/4 (year*)) OR YLL OR YLLs OR ((year*) NEXT/2 (life*) NEXT/1 (lost*)) OR YLD OR YLDs OR ((year*) NEAR/3 (lived) NEAR/3 (disabil*))):ab,ti,kw) AND ('Europe'/exp OR 'Yugoslavia'/de OR 'Israel'/de OR 'European Union'/de OR 'European'/de OR 'EU citizen'/de OR (europ* OR austria* OR belgium OR belgian* OR Denmark OR danish OR france OR french* OR german* OR ireland OR irish* OR italy OR italian* OR luxemb* OR netherlands OR dutch OR norway OR sweden OR switzerland OR swiss OR united-kingdom OR albania OR armenia OR bosnia* OR herzegovin* OR bulgar* OR croatia* OR cyprus OR czechoslovakia* OR estonia* OR finland OR georgia OR greece OR hungar* OR iceland* OR israel* OR kosov* OR latvia* OR lithuan* OR macedoni* OR malta OR montenegr* OR poland OR polish OR portug* OR romani* OR rumani* OR serbi* OR slovak* OR sloven* OR spain* OR spanish OR turkey* OR mediterran* OR czech* OR england* OR UK OR scotland OR wales OR britain* OR holland* OR scandinav* OR nordic-countr* OR yugoslov* OR baltic* OR flander* OR wallon* OR benelux* OR greek* OR andorra* OR azerbaijan* OR belarus* OR byelarus* OR byelorus* OR white-russia* OR monaco* OR moldova* OR moldovia* OR russian-federat* OR san-marin* OR ukrain*):ab,ti,kw)

**Medline**

((DALY OR DALYs OR ((disabil*) ADJ4 (adjust*) ADJ4 (life*) ADJ4 (year*)) OR YLL OR YLLs OR ((year*) ADJ2 (life*) ADJ (lost*)) OR YLD OR YLDs OR ((year*) ADJ3 (lived) ADJ3 (disabil*))).ab,ti,kf.) AND (exp Europe/ OR Yugoslavia/ OR Israel/ OR European Union/ OR (europ* OR austria* OR belgium OR belgian* OR Denmark OR danish OR france OR french* OR german* OR ireland OR irish* OR italy OR italian* OR luxemb* OR netherlands OR dutch OR norway OR sweden OR switzerland OR swiss OR united-kingdom OR albania OR armenia OR bosnia* OR herzegovin* OR bulgar* OR croatia* OR cyprus OR czechoslovakia* OR estonia* OR finland OR georgia OR greece OR hungar* OR iceland* OR israel* OR kosov* OR latvia* OR lithuan* OR macedoni* OR malta OR montenegr* OR poland OR polish OR portug* OR romani* OR rumani* OR serbi* OR slovak* OR sloven* OR spain* OR spanish OR turkey* OR mediterran* OR czech* OR england* OR UK OR scotland OR wales OR britain* OR holland* OR scandinav* OR nordic-countr* OR yugoslov* OR baltic* OR flander* OR wallon* OR benelux* OR greek* OR andorra* OR azerbaijan* OR belarus* OR byelarus* OR byelorus* OR white-russia* OR monaco* OR moldova* OR moldovia* OR russian-federat* OR san-marin* OR ukrain*).ab,ti,kf.)

**Cochrane**

((DALY OR DALYs OR ((disabil*) NEAR/4 (adjust*) NEAR/4 (life*) NEAR/4 (year*)) OR YLL OR YLLs OR ((year*) NEXT/2 (life*) NEXT/1 (lost*)) OR YLD OR YLDs OR ((year*) NEAR/3 (lived) NEAR/3 (disabil*))):ab,ti) AND ((europ* OR austria* OR belgium OR belgian* OR Denmark OR danish OR france OR french* OR german* OR ireland OR irish* OR italy OR italian* OR luxemb* OR netherlands OR dutch OR norway OR sweden OR switzerland OR swiss OR united-kingdom OR albania OR armenia OR bosnia* OR herzegovin* OR bulgar* OR croatia* OR cyprus OR czechoslovakia* OR estonia* OR finland OR georgia OR greece OR hungar* OR iceland* OR israel* OR kosov* OR latvia* OR lithuan* OR macedoni* OR malta OR montenegr* OR poland OR polish OR portug* OR romani* OR rumani* OR serbi* OR slovak* OR sloven* OR spain* OR spanish OR turkey* OR mediterran* OR czech* OR england* OR UK OR scotland OR wales OR britain* OR holland* OR scandinav* OR nordic-countr* OR yugoslov* OR baltic* OR flander* OR wallon* OR benelux* OR greek* OR andorra* OR azerbaijan* OR belarus* OR byelarus* OR byelorus* OR white-russia* OR monaco* OR moldova* OR moldovia* OR russian-federat* OR san-marin* OR ukrain*):ab,ti)

**Web of Science**

TS=(((DALY OR DALYs OR ((disabil*) NEAR/4 (adjust*) NEAR/4 (life*) NEAR/4 (year*)) OR YLL OR YLLs OR ((year*) NEAR/2 (life*) NEAR/1 (lost*)) OR YLD OR YLDs OR ((year*) NEAR/2 (lived) NEAR/2 (disabil*)))) AND ((europ* OR austria* OR belgium OR belgian* OR Denmark OR danish OR france OR french* OR german* OR ireland OR irish* OR italy OR italian* OR luxemb* OR netherlands OR dutch OR norway OR sweden OR switzerland OR swiss OR united-kingdom OR albania OR armenia OR bosnia* OR herzegovin* OR bulgar* OR croatia* OR cyprus OR czechoslovakia* OR estonia* OR finland OR georgia OR greece OR hungar* OR iceland* OR israel* OR kosov* OR latvia* OR lithuan* OR macedoni* OR malta OR montenegr* OR poland OR polish OR portug* OR romani* OR rumani* OR serbi* OR slovak* OR sloven* OR spain* OR spanish OR turkey* OR mediterran* OR czech* OR england* OR UK OR scotland OR wales OR britain* OR holland* OR scandinav* OR nordic-countr* OR yugoslov* OR baltic* OR flander* OR wallon* OR benelux* OR greek* OR andorra* OR azerbaijan* OR belarus* OR byelarus* OR byelorus* OR white-russia* OR monaco* OR moldova* OR moldovia* OR russian-federat* OR san-marin* OR ukrain*)))

**Google Scholar**

"disability adjusted life years" europe|france|germany|italy|netherlands|norway|sweden|switzerland|"united kingdom"|finland|greece|hungaria|israel|poland|portugal|romania|spain|turkey|england|britain|scandinavia

**Grey literature search and websites of targeted national public health agencies**

**Box 3:** List of grey literature search engines and websites of European public health agencies

*Grey literature search engines*

| - OpenGrey: <http://www.opengrey.eu/> | - **CABDirect:** <http://www.cabdirect.org/> |
| --- | --- |
| - OAIster: <http://oaister.worldcat.org/> | - **WHO:** <https://www.who.int/> |

*Websites of targeted national public health agencies*

| - Albania: [www.ishp.gov.al](http://www.ishp.gov.al) | - **Malta:** [www.deputyprimeminister.gov.mt/](http://www.deputyprimeminister.gov.mt/) |
| --- | --- |
| - Andorra: <https://www.salut.ad/> | - **Montenegro:** [www.ijzcg.me/](http://www.ijzcg.me/) |
| - Austria: <https://goeg.at/> | - **Netherlands:** [www.rivm.nl](http://www.rivm.nl/) |
| - Belarus: <http://minzdrav.gov.by/en/> | - **North Macedonia:** [www.iph.mk](http://www.iph.mk/) |
| - Belgium: [www.sciensano.be/en](http://www.sciensano.be/en) | - **Norway:** [www.fhi.no](http://www.fhi.no/) |
| - Bulgaria: <http://ncpha.government.bg> | - **Poland:** [www.pzh.gov.pl](http://www.pzh.gov.pl/) |
| - Cyprus: <https://www.moh.gov.cy/> | - **Portugal:** [www.ihmt.unl.pt](http://www.ihmt.unl.pt) |
| - Czech Republic: [www.szu.cz](http://www.szu.cz) | - **Republic of Moldova:** [www.msmps.gov.md/](http://www.msmps.gov.md/) |
| - Denmark: [www.si-folkesundhed.dk](http://www.si-folkesundhed.dk) | - **Romania:** [www.insp.gov.ro/](https://www.insp.gov.ro/) |
| - Estonia: [www.tai.ee](http://www.tai.ee) | - **Russian Federation:** [www.minzdrav.gov.ru](http://www.minzdrav.gov.ru) |
| - Finland: [www.thl.fi/en/](http://www.thl.fi/en/) | - **Serbia:** [www.batut.org.rs/english.html](http://www.batut.org.rs/english.html) |
| - France: [www.santepubliquefrance.fr](http://www.santepubliquefrance.fr) | - **Slovakia:** [www.uvzsr.sk/en/](http://www.uvzsr.sk/en/) |
| - Germany: [www.rki.de](http://www.rki.de/EN) | - **Slovenia:** [www.nijz.si](http://www.nijz.si/) |
| - Greece: [www.pasteur.gr/en/public-health/](http://www.pasteur.gr/en/public-health/) | - **Spain:** [www.isciii.es](http://www.isciii.es/) |
| - Hungary: [www.nnk.gov.hu/](http://www.nnk.gov.hu/) | - **Sweden:** [www.folkhalsomyndigheten.se](http://www.folkhalsomyndigheten.se/) |
| - Iceland: [www.landlaeknir.is](http://www.landlaeknir.is/) | - **Switzerland:** [www.bag.admin.ch/bag/de/home.html](http://www.bag.admin.ch/bag/de/home.html) |
| - Ireland: [www.publichealth.ie](http://www.publichealth.ie/) | - **Turkey:** [www.saglik.gov.tr/?_Dil=2](http://www.saglik.gov.tr/?_Dil=2) |
| - Israel: [www.gov.il/](http://www.gov.il/) | - **Ukraine:** <https://en.moz.gov.ua/> |
| - Italy: [www.iss.it/](http://www.iss.it/)  - **Latvia:** [www.rsu.lv/en/institute-public-health](https://www.rsu.lv/en/institute-public-health)  **- Lithuania:** <https://sam.lrv.lt/en/>  **- Luxembourg:** [www.lih.lu/](https://www.lih.lu/) | - **United Kingdom:** [www.gov.uk/government/organisations/public-health-england](https://www.gov.uk/government/organisations/public-health-england) |

**Definitions for the systematic review extraction form categories**

| General Information | PMID | A unique identifier number which assigned to a specific reference/article in the PubMed website. |
| --- | --- | --- |
|  | Journal | The name of the journal that published the selected/eligible BoD study. |
|  | Title | The full title of the selected/eligible BoD study. |
|  | Author(s) | List of author(s) with the use of Vancouver style. |
|  | Year | The year that the selected/eligible BoD study was published. |
|  | Objective(s) | The main objective(s) of the BoD study. |
|  | Funding body  *(optional)* | A type of funding source, i.e.: government, institutional administrators, private industry, foundations, professional organisation, etc. |
|  | Language | The written language of the BoD study. |
| Study characteristics  Study characteristics  *(continued)* | Cause of ill health | A single disease related to communicable or non-communicable disease or injury or an aggregation of diseases and injuries.  i.e., Non-communicable diseases (NCDs) [Yes/No]; Communicable diseases (CDs) [Yes/No]; Injuries [Yes/No]; Overall |
|  | GBD study | Global BoD or National BoD? |
|  | Reference population | Population whose health causes during some period of time is the source of the study data (specific multi-country; optional) |
|  | Reference year | The year for which an estimate of incidence/prevalence/BoD is reported. |
|  | Stratification | The specific cause(s), and/or disease(s), and/or risk factor(s) related to mortality or disability indicators stratified for each year, age, and sex. [Yes/No] |
| Data input sources mortality/YLL | Data source mortality/YLL | *Were data sources that were used to derive mortality/YLL data specified by the authors?* [Yes/No]  *Relevant approaches for mortality data:*  National statistics, disease registries, registry of death, survey data, vital registration systems, verbal autopsies, sample death registration systems, published literature, etc. |
| Data input sources incidence/prevalence/YLD | Data source incidence/prevalence/YLD | *Were data sources that were used to derive incidence/prevalence/YLD data specified by the authors?* [Yes/No]  *Relevant approaches for morbidity data:*  Published literature, disease registries, routine administrative and survey datasets, surveillance systems, health facility data, etc |
| Data adjustments  Data adjustments  *(continued)* | Mortality/YLL: data adjustment | [Yes/No] |
|  | Mortality/YLL: data integration | *Were multiple data sources integrated to arrive at the mortality/YLL data?* [Yes/No] |
|  | Incidence/prevalence/YLD: data adjustment | [Yes/No] |
|  | Incidence/prevalence/YLD: data integration | *Were multiple data sources integrated to arrive at the incidence/prevalence/YLD data?* [Yes/No] |
|  | Internal consistency | *Were adjustments made to ensure that the sum of cause‐specific mortality or impairments equals all‐cause mortality or impairments?*  Also known as the mortality (or morbidity) envelope [Yes/No] |
|  | Use of DisMod | DisMod is s a software tool that may be used to check the consistency of estimates of incidence, prevalence, duration and case fatality for diseases.  *Did the authors mention the use of DisMoD?* [Yes/No] |
| DALY method  DALY method  *(continued)*  DALY method  *(continued)* | Perspective of YLD estimates | - *Prevalence-based* perspective takes point prevalence measures of disability, adjusted for seasonal variation. - *Incidence-based* perspective captures the BoD in new diagnostic cases during a reference time-period and links all possible sequelae in future through an outcome tree or disease progression model. |
|  | Life expectancy for YLL | The life table that was used to assess YLL  *Relevant life-tables*:  Aspirational life-tables (i.e., WHO or GBD standard life table), or country-specific life tables |
|  | Disease model | A disease model is a causal chain of a disease that describes health states and their transition probabilities over time.  *Did the authors report the disease model that they have used to assess BoD?* [Yes/No] |
|  | DW: source | The source of the set(s) of disability weights (DWs) that were used to assess YLD.  *Relevant sources*: GBD DWs, Dutch DWs, etc. |
|  | DW: elicitation method  (only if study developed own DWs) | Methods for eliciting health state valuations  *Relevant methods*: Visual Analogue Scale (VAS), Person Trade-Off (PTO), Time Trade-Off (TTO), etc. |
|  | DW: panel of judges  (only if study developed own DWs) | The panel of judges whose preferences were obtained to assess DWs  *Relevant panel composition*: Medical experts, healthcare professionals, policymakers, patients/people with disabilities, patients’ families, etc. |
|  | DW: severity distribution | The proportion of cases with e.g., mild, moderate or severe health state^1^ of a specific outcome for which separate DWs are available. *Was a severity distribution used/reported by the authors?* [Yes/No] / [Global/National]  ^1^: a health state reflects a combination of signs or symptoms that result in a certain amount of health loss |
|  | Multi-morbidity adjustment (YLD calculation) | Adjustment of YLD data for multi-morbidity^2^ [Yes/No]  ^2^: co-occurrence of two or more morbid conditions in the same individual |
|  | Methods for YLD multi-morbidity adjustment | Approach(es) used to deal with the impact of multi-morbidity e.g., Standard simulation method, Multiplicative approach, etc |
|  | Social weighting:  age weighting | By incorporating age-weighting into DALY implies that the value of life depends on age; a lower weight of healthy life years lived is given at younger and at older ages – known as ‘non-uniform DALY’ [Yes/No] |
|  | Social weighting:  time discounting | Time-discounting discounts future years of healthy life lived using a rate of 3% or an alternative set of 0% [Yes/No] |
|  | Social weighting: discounting rate | ‘*uniform DALY’*; age-weighting and 3% time-discounting rate; ‘*non-uniform DALY’*; no age-weighting, no time-discounting rate; ‘*age-weighting DALY’*; age-weighting, no time-discounting rate; ‘*time-discounting DALY’*; no age-weighting, 3% time-discounting rate [%] |
| Uncertainty  Uncertainty  *(continued)* | Uncertainty analysis | An estimation of range or distribution of uncertainty in estimates based on an assessment of the uncertainty or confidence intervals for all data and parameter inputs  [Yes/No] |
|  | Uncertainty analysis: method | *Relevant methods of uncertainty in DALY calculations:*  Parameter uncertainty, Structural or model uncertainty, Methodological uncertainty, qualitative assessment |
|  | Sensitivity analysis | Analysis of how the impact of uncertainties of one or more input variables can lead to uncertainties in data inputs or assumptions. (e.g., showing a Tornado plot); [Yes/No] |
|  | Scenario analysis | The current or future disease burden is compared with the BoD if one element is changed (e.g., life expectancy, DW or severity distribution) [Yes/No] |
|  | Scenario analysis: element changed | Element that was changed for the scenario analysis  (e.g., life expectancy, DW or severity distribution) |
| BoD: burden of disease; DALY: disability adjusted life years; DW: disability weight; GBD: Global Burden of Disease; PMID: PubMed Identifier; YLD: years lived with disability; YLL: years of life lost due to premature mortality; WHO: World Health Organisation.  * Please note that the disease burden items in the boxes filled with grey colour were discussed in this systematic review. | | |

**Reference list of included European burden of disease studies**

| Consecutive number | Author(s) | Year | Title | Country(s) / Region |
| --- | --- | --- | --- | --- |
|  |  |  |  |  |
| 1 | Agardh et al | 2018 | Hälsoutvecklingen i Stockholm bättre än i övriga landet - Jämförelse av hälsoläget utifrån globala Sjukdomsbördeprojektet | Sweden |
| 2 | Akgün et al | 2007 | Estimating mortality and causes of death in Turkey: methods, results and policy implications | Turkey |
| 3 | Araujo et al | 2016 | Regional variation in coronary heart disease mortality trends in Portugal, 1981-2012 | Portugal (Alentejo, Azores, Algarve, Center, Lisbon, Madeira and North) |
| 4 | Atalab et al | 2020 | The global, regional, and national burden of inflammatory bowel disease in 195 countries and territories, 1990–2017: a systematic analysis for the Global Burden of Disease Study 2017 | Global |
| 5 | Baranne & Falissard | 2018 | Global burden of mental disorders among children aged 5–14 years | Global |
| 6 | Barchuk et al | 2018 | Breast and cervical cancer incidence and mortality trends in Russia 1980-2013 | Russia Federation |
| 7 | Barker-Collo et al | 2015 | Sex Differences in Stroke Incidence, Prevalence, Mortality and Disability-Adjusted Life Years: Results from the Global Burden of Disease Study 2013 | Global |
| 8 | Beghi et al | 2019 | Global, regional, and national burden of epilepsy, 1990–2016: a systematic analysis for the Global Burden of Disease Study 2016 | Global |
| 9 | Bikbov et al | 2014 | Mortality landscape in the Global Burden of Diseases, Injuries and Risk Factors Study | Global |
| 10 | Bikbov et al | 2020 | Global, regional, and national burden of chronic kidney disease, 1990–2017: a systematic analysis for the Global Burden of Disease Study 2017 | Global |
| 11 | Bowie et al | 1997 | Estimating the burden of disease in an English region | South and West Region of England |
| 12 | Brustugun et al | 2014 | Years of life lost as a measure of cancer burden on a national level | Norway |
| 13 | Burazeri et al | 2014 | National Health Report: Health Status of the Albanian Population | Albania |
| 14 | Burnet et al | 2005 | Years of life lost (YLL) from cancer is an important measure of population burden – and should be considered when allocating research funds | East Anglia |
| 15 | Catala-Lopez et al | 2013 | Burden of disease assessment with summary measures of population health for the Region of Valencia, Spain: a population-based study | Spain (Region of Valencia) |
| 16 | Catala-Lopez et al | 2013 | Carga de enfermedad en adolescentes y jóvenes en España | Spain |
| 17 | Catala-Lopez et al | 2014 | The national burden of cerebrovascular diseases in Spain: a population-based study using disability-adjusted life years | Spain |
| 18 | Cortes Garcia et al | 2004 | Carga de enfermedad de la población española menor de 15 años para el año 1999 | Spain |
| 19 | Cowan et al | 2018 | Global Burden of Multiple Myeloma: A Systematic Analysis for the Global Burden of Disease Study 2016 | Global |
| 20 | Cuadras Andreu & Rovira Ricart | 2014 | Carga de enfermedad en la comunidad autónoma de Cataluña utilizando años de vida ajustados por discapacidad (2005-2010) | Spain (Catalonia) |
| 21 | Cubo et al | 2005 | Burden of Disease Related to Parkinson’s Disease in Spain in the Year 2000 | Spain |
| 22 | Darba et al | 2015 | Disability-adjusted life years lost due to diabetes in France, Italy, Germany, Spain, and the United Kingdom: a burden of illness study | France, Italy, Germany, Spain, and United Kingdom |
| 23 | Dirac et al | 2020 | The global, regional, and national burden of gastro-oesophageal reflux disease in 195 countries and territories, 1990–2017: a systematic analysis for the Global Burden of Disease Study 2017 | Global |
| 24 | Direção-Geral da Saúde | 2018 | Portugal: The Nation's Health 1990-2016; an overview of the Global Burden of Disease Study 2016 Results | Portugal |
| 25 | Dodhia & Phillips | 2008 | Measuring burden of disease in two inner London boroughs using Disability Adjusted Life Years | London (boroughs of Lambeth and Southwark) |
| 26 | Donnay et al | 2013 | Carga de la enfermedad atribuible al hipotiroidismo subclínico en la población espanola | Spain |
| 27 | Effertz & Mann | 2013 | The burden and cost of disorders of the brain in Europe with the inclusion of harmful alcohol use and nicotine addiction | Europe |
| 28 | Essink-Bot et al | 2002 | Cross-national comparability of burden of disease estimates: the European Disability Weights Project | Denmark; England and Wales; France; The Netherlands; Spain; Sweden |
| 29 | Feigin et al | 2019 | Global, regional, and national burden of neurological disorders, 1990–2016: a systematic analysis for the Global Burden of Disease Study 2016 | Global |
| 30 | Fernandez de Larrea-Baz et al | 2009 | Burden of disease due to cancer in Spain | Spain |
| 31 | Fernandez de Larrea-Baz | 2015 | Disability-adjusted Life Years Lost to Ischemic Heart Disease in Spain | Spain |
| 32 | Fitzmaurice et al | 2019 | Global, Regional, and National Cancer Incidence, Mortality, Years of Life Lost, Years Lived With Disability, and Disability-Adjusted Life-Years for 29 Cancer Groups, 1990 to 2017: A Systematic Analysis for the Global Burden of Disease Study | Global |
| 33 | Force et al | 2019 | The global burden of childhood and adolescent cancer in 2017: an analysis of the Global Burden of Disease Study 2017 | Global |
| 34 | Francescutti et al | 2005 | The impact of stroke in Italy: first step for a National Burden of Disease Study | Italy |
| 35 | Ganczakl et al | 2020 | Changes in disease burden in Poland between 1990-2017 in comparison with other Central European countries: A systematic analysis for the Global Burden of Disease Study 2017 | Poland |
| 36 | Genova-Maleras et al | 2011 | Burden of disease in the elderly population in Spain | Spain |
| 37 | Genova-Maleras et al | 2012 | Measuring the burden of disease and injury in Spain using disability-adjusted life years: an updated and policy-oriented overview | Spain |
| 38 | Gore et al | 2011 | Global burden of disease in young people aged 10-24 years: a systematic analysis | Global |
| 39 | Gouveia et al | 2019 | The current and future burden of heart failure in Portugal | Portugal |
| 40 | Grant et al | 2015 | The Scottish Burden of Disease (SBoD) study; Age–gender report | Scotland |
| 41 | Grant et al | 2017 | The burden of disease is generally greater in more deprived areas, and that burden is more likely to be fatal | Scotland |
| 42 | Haro et al | 2014 | The burden of disease in Spain: results from the global burden of disease study 2010 | Spain |
| 43 | Hay et al | 2014 | The global burden of skin disease in 2010: an analysis of the prevalence and impact of skin conditions | Global |
| 44 | Hay et al | 2017 | Global, regional, and national disability-adjusted life-years (DALYs) for 333 diseases and injuries and healthy life expectancy (HALE) for 195 countries and territories, 1990–2016: a systematic analysis for the Global Burden of Disease Study 2016 | Global |
| 45 | Henriques et al | 2017 | Disability-adjusted life years lost due to ischemic heart disease in mainland Portugal, 2013 | Portugal (Alentejo, Algarve, Center, Lisbon, North) |
| 46 | Hilderink et al | 2016 | Accounting for multimorbidity can affect the estimation of the Burden of Disease: a comparison of approaches | Netherlands |
| 47 | Hollestein et al | 2014 | Burden of disease caused by keratinocyte cancer has increased in The Netherlands since 1989 | Netherlands |
| 48 | Hollinghurst et al | 2000 | Estimating the "avoidable" burden of disease by Disability Adjusted Life Years (DALYs) | UK |
| 49 | Holterhues et al | 2013 | Burden of disease due to cutaneous melanoma has increased in the Netherlands since 1991 | Netherlands |
| 50 | Iovu & Breaz | 2019 | The prevalence and burden of mental and substance use disorders in Romania: Findings from the Global Burden of Disease Study 2016 | Romania |
| 51 | James et al | 2018 | Global, regional, and national incidence, prevalence, and years lived with disability for 354 diseases and injuries for 195 countries and territories, 1990–2017: a systematic analysis for the Global Burden of Disease Study 2017 | Global |
| 52 | Jankovic et al | 2007 | The burden of disease and injury in Serbia | Serbia, Serbia and Montenegro |
| 53 | Jayatilleke et al | 2011 | Burden of disease due to cancer in England and Wales | England & Wales |
| 54 | Karelson | 2016 | Eesti rahvastiku tervisekaotus 2013. Aastal | Estonia |
| 55 | Karimkhani et al | 2017 | The global burden of melanoma: results from the Global Burden of Disease Study 2015 | Global |
| 56 | Karimkhani et al | 2017 | Global Skin Disease Morbidity and Mortality: An Update From the Global Burden of Disease Study 2013 | Global |
| 57 | Kassebaum et al | 2016 | Global, regional, and national disability-adjusted life-years (DALYs) for 315 diseases and injuries and healthy life expectancy (HALE), 1990-2015: a systematic analysis for the Global Burden of Disease Study 2015 | Global |
| 58 | Kassebaum et al | 2017 | Global, Regional, and National Prevalence, Incidence, and Disability-Adjusted Life Years for Oral Conditions for 195 Countries, 1990-2015: A Systematic Analysis for the Global Burden of Diseases, Injuries, and Risk Factors | Global |
| 59 | Kaufmann et al | 2020 | 60/30: 60% of the Morbidity-Associated Multiple Sclerosis Disease Burden Comes From the 30% of Persons With Higher Impairments | Switzerland |
| 60 | Kiadaliri et al | 2018 | Burden of rheumatoid arthritis in the Nordic region, 1990–2015: a comparative analysis using the Global Burden of Disease Study 2015 | Nordic Region (Denmark, Finland, Greenland, Iceland, Norway, Sweden) |
| 61 | Kiadaliri et al | 2018 | Burden of gout in the Nordic region, 1990–2015: findings from the Global Burden of Disease Study 2015 | Nordic Region (Denmark, Finland, Greenland, Iceland, Norway, Sweden) |
| 62 | Kiadaliri et al | 2018 | High and rising burden of hip and knee osteoarthritis in the Nordic region, 1990-2015 | Nordic region (Denmark, Finland, Greenland, Iceland, Norway, Sweden) |
| 63 | Kinge et al | 2017 | Economic losses and burden of disease by medical conditions in Norway | Norway |
| 64 | Kissimova-Skarbek | 2016 | Approaches to Disease Burden Measurement: Disability-Adjusted Life Years (DALYs) Globally and in Poland, and National Income Lost Due to Disease in Poland, 1990–2015 | Poland |
| 65 | Kissimova-Skarbek et al | 2001 | Ocena ekonomicznego obciążenia cukrzyca społeczeństwa Polski | Poland |
| 66 | Knape & Dhein | 2020 | Changes in causes and age of death in an eastern German county over a period of 14 years. Comparison of rural and urban populations. Focus on COPD and ischemic heart disease | Germany (Thuringia) |
| 67 | Knudsen et al | 2016 | Sykdomsbyrde i Norge 1990-2013 | Norway |
| 68 | Knudsen et al | 2017 | Sykdomsbyrde i Norge 2015; Resultater fra Global Burden of Diseases, Injuries, and Risk Factors Study 2015 (GBD 2015) | Norway |
| 69 | Knudsen et al | 2019 | Life expectancy and disease burden in the Nordic countries: results from the Global Burden of Diseases, Injuries, and Risk Factors Study 2017 | Nordic countries (Denmark, Finland, Iceland, Norway, Sweden) and Greenland |
| 70 | Kobyakova et al | 2014 | Динамика числа потерянных лет жизни (DALY) в результате преждевременной смертности детей в возрасте 0–17 лет в Томской области в 2008–2012 гг (Dynamics of Disability Adjusted Life Years (DALY) as a Result of Premature Mortality of Children at the Age of 0–17 Years in Tomsk Region in 2008–2012) | Tomsk Region of Russian |
| 71 | Koko et al | 2015 | Epidemiology of Rheumatoid Arthritis in Southern Albania | Southern Albania (Gjirokaster) |
| 72 | Krishnamurthi et al | 2015 | Stroke Prevalence, Mortality and Disability-Adjusted Life Years in Children and Youth Aged 0-19 Years: Data from the Global and Regional Burden of Stroke 2013 | Global |
| 73 | Krishnamurthi et al | 2015 | Stroke Prevalence, Mortality and Disability-Adjusted Life Years in Adults Aged 20-64 Years in 1990-2013: Data from the Global Burden of Disease 2013 Study | Global |
| 74 | Kruijshaar & Barendregt | 2004 | The breast cancer related burden of morbidity and mortality in six European countries: the European Disability Weights project | Denmark; England and Wales; France; The Netherlands; Spain; Sweden |
| 75 | Kyu et al | 2018 | Global, regional, and national disability-adjusted life-years (DALYs) for 359 diseases and injuries and healthy life expectancy (HALE) for 195 countries and territories, 1990-2017: a systematic analysis for the Global Burden of Disease Study 2017 | Global |
| 76 | Lai et al | 2004 | Haiguskoormuse tõttu kaotatud eluaastad Eestis: seosed riskifaktoritega ja riskide vähendamise kulutõhusus | Estonia |
| 77 | Lai et al | 2006 | Maakondlik haiguskoormus Eestis 2000 – 2004 | Estonia |
| 78 | Lai & Kohler | 2009 | Burden of Disease of Estonian population | Estonia (Hiiu, Saare, Rapla, Ida-Viru, Põlva, Võru, Harju, Tartu, Lääne, Pärnu, Viljandi, Järva, Lääne-Viru, Jõgeva, Valga) |
| 79 | Lai et al | 2009 | Measuring burden of disease in Estonia to support public health policy | Estonia |
| 80 | Lokkerbol et al | 2013 | Non-fatal burden of disease due to mental disorders in the Netherlands | Netherlands |
| 81 | Lozano et al | 2012 | Global and regional mortality from 235 causes of death for 20 age groups in 1990 and 2010: a systematic analysis for the Global Burden of Disease Study 2010 | Global |
| 82 | Lapostolle et al | 2007 | Sensitivity analysis in summary measure of population health in France | France |
| 83 | Lara et al | 2015 | La carga de la enfermedad en Espana 2010: trastornos neurológicos, mentales y re: trastornos neurológicos, mentales y relacionados con el consumo de sustancias | Spain |
| 84 | Laursen et al | 2019 | Cause-specific life years lost among persons diagnosed with schizophrenia: Is it getting better or worse? | Denmark |
| 85 | Lekhan & Kriachkova | 2019 | The system of measures to improve the health of the population of Ukraine based on the analysis of the global burden of diseases and its risk factors | Ukraine |
| 86 | Leonardi et al | 2002 | The global burden of epilepsy | Global |
| 87 | Leonardi et al | 2005 | The global burden of migraine: measuring disability in headache disorders with WHO's Classification of Functioning, Disability and Health (ICF). | Global |
| 88 | Lewis & Torgerson | 2017 | The current and future burden of late-onset dementia in the United Kingdom: Estimates and interventions | UK (England & Wales) |
| 89 | Li et al | 2020 | Trends and risk factors of mortality and disability adjusted life years for chronic respiratory diseases from 1990 to 2017: systematic analysis for the Global Burden of Disease Study 2017 | Global |
| 90 | Ljung et al | 2005 | Socioeconomic differences in the burden of disease in Sweden | Sweden |
| 91 | Logroscino et al | 2018 | Global, regional, and national burden of motor neuron diseases 1990–2016: a systematic analysis for the Global Burden of Disease Study 2016 | Global |
| 92 | Lopez et al | 2006 | Global and regional burden of disease and risk factors, 2001: systematic analysis of population health data | Global |
| 93 | López-Jurado et al | 2008 | Mortality and hospital utilization due to breast cancer in Extremadura, Spain (2002-2004) | Spain (Extremadura) |
| 94 | Lunevicius et al | 2015 | National burden of colorectal cancer in Lithuania and the ranking of Lithuania within the 45 European nations | Lithuania |
| 95 | Machado et al | 2011 | Carga Global da Doença na região Norte de Portugal | Northern region of Portugal |
| 96 | Maertens de Noordhout et al | 2018 | Changes in health in Belgium, 1990–2016: a benchmarking analysis based on the global burden of disease 2016 study | Belgium |
| 97 | Mariotti et al | 2003 | Years of life lost due to premature mortality in Italy | Italy |
| 98 | Mazzotti et al | 2019 | Years of Life Lost for Older Patients After Colorectal Cancer Diagnosis | Netherlands |
| 99 | Melse & Kramers | 1998 | Berekeningen van de ziektelast in Nederland. Achtergronddocument bij VTV-1997 deel III, hoofdstuk 7 | Netherlands |
| 100 | Melse et al | 2000 | A national burden of disease calculation: Dutch disability-adjusted life-years. Dutch Burden of Disease Group | Netherlands |
| 101 | Mesalles-Naranjo et al | 2018 | Trends and inequalities in the burden of mortality in Scotland 2000–2015 | Scotland |
| 102 | Minicuci et al | 2011 | Chronic obstructive pulmonary disease: the Disability Adjusted Life Years in northern Italy | Italy |
| 103 | Mokdad et al | 2016 | Global burden of diseases, injuries, and risk factors for young people's health during 1990-2013: a systematic analysis for the Global Burden of Disease Study 2013 | Global |
| 104 | Monasta et al | 2019 | Italy’s health performance, 1990–2017: findings from the Global Burden of Disease Study 2017 | Italy |
| 105 | Moradi et al | 2006 | Neuropsykiatriska sjukdomar och hjärt–kärlsjukdomar dominerar | Sweden |
| 106 | Murray et al | 2012 | Disability-adjusted life years (DALYs) for 291 diseases and injuries in 21 regions, 1990-2010: a systematic analysis for the Global Burden of Disease Study 2010 | Global |
| 107 | Murray et al | 2013 | UK health performance: findings of the Global Burden of Disease Study 2010 | United Kingdom |
| 108 | Murray et al | 2015 | Global, regional, and national disability-adjusted life years (DALYs) for 306 diseases and injuries and healthy life expectancy (HALE) for 188 countries, 1990-2013: quantifying the epidemiological transition | Global |
| 109 | Murphy et al | 2017 | Ischaemic heart disease in the former Soviet Union 1990-2015 according to the Global Burden of Disease 2015 Study | Global |
| 110 | Naghavi et al | 2017 | Global, regional, and national age-sex specific mortality for 264 causes of death, 1980–2016: a systematic analysis for the Global Burden of Disease Study 2016 | Global |
| 111 | Nascimento et al | 2018 | Cardiovascular Disease Epidemiology in Portuguese-Speaking Countries: data from the Global Burden of Disease, 1990 to 2016 | Portugal |
| 112 | Newton et al | 2015 | Changes in health in England, with analysis by English regions and areas of deprivation, 1990–2013: a systematic analysis for the Global Burden of Disease Study 2013 | England |
| 113 | Nichols et al | 2019 | Global, regional, and national burden of Alzheimer’s disease and other dementias, 1990–2016: a systematic analysis for the Global Burden of Disease Study 2016 | Global |
| 114 | Nielsen et al | 2004 | Burden of mortality in Greenland--today and tomorrow | Greenland |
| 115 | Olesen & Leonardi | 2003 | The burden of brain diseases in Europe | Europe (EURO A; EURO B; EURO C) |
| 116 | Øverland et al | 2018 | Sykdomsbyrden i Norge 2016; Resultater fra Global Burden of Diseases, Injuries, and Risk Factors Study 2016 (GBD 2016) | Norway |
| 117 | Paik et al | 2020 | Changes in the Global Burden of Chronic Liver Diseases From 2012 to 2017: The Growing Impact of Nonalcoholic Fatty Liver Disease | Global |
| 118 | Pardo et al | 2016 | The Global Burden of Esophageal Cancer: A Disability-Adjusted Life-Year Approach | Global |
| 119 | Patel et al | 2019 | Global, regional, and national burden of brain and other CNS cancer, 1990–2016: a systematic analysis for the Global Burden of Disease Study 2016 | Global |
| 120 | Pereira et al | 2012 | Changing patterns of cardiovascular diseases and cancer mortality in Portugal, 1980-2010 | Portugal |
| 121 | Pishgar et al | 2019 | Global, regional and national burden of testicular cancer, 1990-2016: results from the Global Burden of Disease Study 2016 | Global |
| 122 | Plana-Ripoll et al | 2019 | A comprehensive analysis of mortality-related health metrics associated with mental disorders: a nationwide, register-based cohort study | Denmark |
| 123 | Plass et al | 2014 | Trends in disease burden in Germany: results, implications and limitations of the Global Burden of Disease study | Germany |
| 124 | Powles et al | 2005 | The contribution of leading diseases and risk factors to excess losses of healthy life in eastern Europe: burden of disease study | Europe |
| 125 | Raggi et al | 2019 | Burden of brain disorders in Europe in 2017 and comparison with other non-communicable disease groups | EU-28 countries; Iceland, Norway, Switzerland |
| 126 | Ray Dorsey et al | 2018 | Global, regional, and national burden of Parkinson’s disease, 1990–2016: a systematic analysis for the Global Burden of Disease Study 2016 | Global |
| 127 | Reiner et al | 2019 | Diseases, Injuries, and Risk Factors in Child and Adolescent Health, 1990 to 2017: Findings From the Global Burden of Diseases, Injuries, and Risk Factors 2017 Study | Global |
| 128 | Rommel et al | 2018 | BURDEN 2020 - Burden of disease in Germany at the national and regional level | Germany |
| 129 | Roth et al | 2018 | Global, regional, and national age-sex-specific mortality for 282 causes of death in 195 countries and territories, 1980–2017: a systematic analysis for the Global Burden of Disease Study 2017 | Global |
| 130 | Safiri et al | 2019 | Global, regional and national burden of rheumatoid arthritis 1990–2017: a systematic analysis of the Global Burden of Disease study 2017 | Global |
| 131 | Sanchez-Valle et al | 2008 | Estimating the Burden of Disease for Autism Spectrum Disorders in Spain in 2003 | Spain |
| 132 | Santos et al | 2019 | The state of health in the European Union (EU-28) in 2017: an analysis of the burden of diseases and injuries | EU-28 countries |
| 133 | Santric Milicevic  et al | 2009 | Serbia within the European context: An analysis of premature mortality | Serbia (Kosovo and Metohija were excluded); EURO A; EURO B; EURO C |
| 134 | Schopper et al | 2000 | Estimating the burden of disease in one Swiss canton: what do disability adjusted life years (DALY) tell us? | Swiss canton; Geneva |
| 135 | Sebbag et al | 2019 | The world-wide burden of musculoskeletal diseases: a systematic analysis of the World Health Organization Burden of Diseases Database | Global |
| 136 | Sepanlou et al | 2020 | The global, regional, and national burden of cirrhosis by cause in 195 countries and territories, 1990-2017: a systematic analysis for the Global Burden of Disease Study 2017 | Global |
| 137 | Soerjomataram et al | 2012 | Global burden of cancer in 2008: a systematic analysis of disability-adjusted life-years in 12 world regions | Global |
| 138 | Soriano et al | 2018 | The burden of disease in Spain: Results from the Global Burden of Disease 2016 | Spain |
| 139 | Starodubov et al | 2018 | The burden of disease in Russia from 1980 to 2016: a systematic analysis for the Global Burden of Disease Study 2016 | Russia Federation |
| 140 | Steel et al | 2018 | Changes in health in the countries of the UK and 150 English Local Authority areas 1990-2016: a systematic analysis for the Global Burden of Disease Study 2016 | UK; England; Scotland; Wales; Northern Ireland; 150 English Upper-Tier Local Authorities |
| 141 | Stockton et al | 2016 | The Scottish Burden of Disease (SBoD) study; Deprivation report | Scotland |
| 142 | Tataru et al | 2019 | The Situation of Cervical Cancers in the Context of Female Genital Cancer Clustering and Burden of Disease in Arad County, Romania | Arad country, Romania |
| 143 | Tollanes et al | 2018 | Disease burden in Norway in 2016 | Norway |
| 144 | Tromme et al | 2016 | Melanoma burden by melanoma stage: Assessment through a disease transition model | Belgium |
| 145 | Tsilidis et al | 2016 | Burden of Cancer in a Large Consortium of Prospective Cohorts in Europe | Europe |
| 146 | Tyrovolas et al | 2018 | The burden of disease in Greece, health loss, risk factors, and health financing, 2000–16: an analysis of the Global Burden of Disease Study 2016 | Greece |
| 147 | Ünüvar et al | 2006 | Turkey Burden of Disease Study | Turkey |
| 148 | Ustun et al | 2004 | Global burden of depressive disorders in the year 2000 | Euro A (UK, The Netherlands, Ireland, Czech Republic, Spain, Norway, Germany, France, Italy, Greece, Finland); Euro BI (Turkey, Georgia, Slovakia); Euro B2; Euro C (Russian Federation) |
| 149 | Valent & Zanier | 2015 | A population-based study of the years of life lost in the Friuli Venezia Giulia region, Italy | Italy (Friuli Venezia Giulia) |
| 150 | Villaverde-Hueso  et al | 2007 | Estimating the burden of scleroderma disease in Spain | Spain |
| 151 | Vlajinac et al | 2006 | Burden of Cancer in Serbia | Central Serbia; Vojvodina; Serbia and Montenegro |
| 152 | Vlajinac et al | 2006 | Burden of ischaemic heart disease and cerebrovascular diseases in Serbia without Kosovo and Metohija, 2000 | Serbia, Serbia and Montenegro (without Kosovo and Metohija) |
| 153 | Vlajinac et al | 2008 | Years of life lost due to premature death in Serbia (excluding Kosovo and Metohija) | Serbia (excluding Kosovo and Metohija) |
| 154 | Vondeling et al | 2018 | Burden of early, advanced and metastatic breast cancer in The Netherlands | Netherlands |
| 155 | Vos et al | 2015 | Global, regional, and national incidence, prevalence, and years lived with disability for 301 acute and chronic diseases and injuries in 188 countries, 1990-2013: a systematic analysis for the Global Burden of Disease Study 2013 | Global |
| 156 | Vos et al | 2012 | Years lived with disability (YLDs) for 1160 sequelae of 289 diseases and injuries 1990–2010: a systematic analysis for the Global Burden of Disease Study 2010 | Global |
| 157 | Wallin et al | 2019 | Global, regional, and national burden of multiple sclerosis 1990–2016: a systematic analysis for the Global Burden of Disease Study 2016 | Global |
| 158 | Whiteford et al | 2013 | Global burden of disease attributable to mental and substance use disorders: findings from the Global Burden of Disease Study 2010 | Global |
| 159 | Whiteford et al | 2015 | The global burden of mental, neurological and substance use disorders: an analysis from the Global Burden of Disease Study 2010 | Global |
| 160 | WHO | 2008 | The global burden of disease: 2004 update | Global |
| 161 | Witthaus et al | 1999 | Burden of mortality and morbidity from dementia | Netherlands |
| 162 | Wittchen et al | 2011 | The size and burden of mental disorders and other disorders of the brain in Europe 2010 | Europe |
| 163 | Yang et al | 2004 | Cervical cancer as a priority for prevention in different world regions: an evaluation using years of life lost | Global |

**Data input sources for morbidity and mortality in single-country burden of disease studies (N=96)**

| Consecutive number | Author(s) | Year | Country(s) / Region | Data input sources for mortality | Data input sources for morbidity |
| --- | --- | --- | --- | --- | --- |
| 1 | Agardh et al | 2018 | Sweden | GBD estimates | GBD estimates |
| 2 | Akgün et al | 2007 | Turkey | State Institute of Statistics (SIS; Urban Turkey); Special Mortality and Verbal Autopsy survey (Rural Turkey); Demography and Health Survey; Traffic Police  records/Ministry of Justice | NA |
| 3 | Araujo et al | 2016 | Portugal | Official National Statistics | NA |
| 4 | Barchuk et al | 2018 | Russia Federation | Russian State Cancer Registry | NA |
| 5 | Bowie et al | 1997 | South & West Region of England | 1992 Office of Population Censuses  and Surveys (OPCS) age-specific mortality data | World Bank study |
| 6 | Brustugun et al | 2014 | Norway | Cancer Registry of Norway | NA |
| 7 | Burazeri et al | 2014 | Albania | GBD estimates | GBD estimates |
| 8 | Burnet et al | 2005 | East Anglia | East Anglian Cancer Registry | NA |
| 9 | Catala-Lopez et al | 2013 | Spain | Death registry data | WHO/GBD estimates |
| 10 | Catala-Lopez et al | 2013 | Spain | Vital Statistics from the Spanish  National Statistics Institute | WHO/GBD estimates |
| 11 | Catala-Lopez et al | 2014 | Spain | National Statistics; Spanish Ministry of Economy and Competitiveness; IBERICTUS | IBERICTUS study (literature; other) |
| 12 | Cortes Garcia et al | 2004 | Spain | National Statistics Institute | National epidemiological surveillance network; Hospital morbidity survey; WHO |
| 13 | Cuadras Andreu & Rovira Ricart | 2014 | Spain | Catalonia mortality registry | Tarragona cancer registry |
| 14 | Cubo et al | 2005 | Spain | National Statistics Institute (Instituto Nacional de Estadıstica) | National epidemiological data |
| 15 | Direção-Geral da Saúde | 2018 | Portugal | GBD estimates | GBD estimates |
| 16 | Dodhia & Phillips | 2008 | London | Local mortality data | GBD estimates |
| 17 | Donnay et al | 2013 | Spain | Vital Statistics from the Spanish National Statistics Institute; inpatient mortality records, etc | Morbidity from the Basic Minimum Set of Data of Hospitalization (BMSD-H); hospital discharge records, etc |
| 18 | Fernandez de  Larrea-Baz et al | 2009 | Spain | National Institute of Statistics Spain; National cause-of-death register | Spanish population-based cancer registries, Survival data in Spain from the European Cancer Registries study |
| 19 | Fernandez de Larrea-Baz | 2015 | Spain | Spanish National Institute of Statistics | Spanish Registry of Hospital Discharges (Minimum Basic Data Set); literature |
| 20 | Francescutti et al | 2005 | Italy | National Bureau of Census | Routine database-PBHIS (population-based health information system) of Friuli Venezia-Giulia |
| 21 | Ganczakl et al | 2020 | Poland | National registrations of causes of deaths; health surveys; infectious surveillance; hospitalization data; demographic censuses; scientific literature; epidemiological reports | GBD estimates |
| 22 | Genova-Maleras et al | 2011 | Spain | Vital Statistics from the Spanish National Statistics Institute | WHO/GBD estimates |
| 23 | Genova-Maleras et al | 2012 | Spain | Spanish National Statistics Institute (Instituto Nacionalde Estadıstica) | WHO/GBD estimates |
| 24 | Gouveia et al | 2019 | Portugal | European Detailed Mortality Database of the WHO | EPICA epi data |
| 25 | Grant et al | 2015 | Scotland | Scottish register of deaths | Routine administrative and survey datasets, largely patient-level records from health service activity |
| 26 | Grant et al | 2017 | Scotland | Scottish register of deaths | Routine administrative and survey datasets, largely patient-level records from health service activity |
| 27 | Haro et al | 2014 | Spain | GBD estimates | GBD estimates |
| 28 | Henriques et al | 2017 | Portugal | Statistics Portugal | National population-based epidemiological studies (direct method, Standard European Population) |
| 29 | Hilderink et al | 2016 | Netherlands | NA | Dutch Public Health Status and Forecasts report (PHSF), GP registration databases, Netherlands cancer registries |
| 30 | Hollestein et al | 2014 | Netherlands | Statistics Netherlands (CBS) | Netherlands Cancer Registry, hospital discharge |
| 31 | Hollinghurst et al | 2000 | United Kingdom | Office of Population, Censuses and Surveys (OPCS) UK | Epidemiological survey data and routinely collected treatment data |
| 32 | Holterhues et al | 2013 | Netherlands | Statistics Netherlands (CBS) | Netherlands Cancer Registry |
| 33 | Iovu & Breaz | 2019 | Romania | GBD estimates | GBD estimates |
| 34 | Jankovic et al | 2007 | Serbia, Serbia and Montenegro | Serbian Office of Statistics 'mortality database' | Disease registers, routine databases, and epidemiological studies |
| 35 | Jayatilleke et al | 2011 | England & Wales | Office for National Statistics (ONS) | Cancer registrations data from Office for National Statistics (ONS), Welsh cancer intelligence and surveillance unit |
| 36 | Karelson | 2016 | Estonia | Vital registration of Statistics Estonia | Estonian Health Insurance Fund database |
| 37 | Kaufmann et al | 2020 | Switzerland | Swiss Multiple Sclerosis Registry; Swiss mortality registry | Swiss epi data |
| 38 | Kinge et al | 2017 | Norway | GBD estimates | GBD estimates |
| 39 | Kissimova-Skarbek | 2016 | Poland | GBD estimates | GBD estimates |
| 40 | Kissimova-Skarbek  et al | 2001 | Poland | Vital registration system | Published literature |
| 41 | Knape & Dhein | 2020 | Germany | Thüringer Landesamt für Statistik (Statistisches Jahrbuch Thüringen 2015); Statistisches Bundesamt | NA |
| 42 | Knudsen et al | 2016 | Norway | GBD estimates | GBD estimates |
| 43 | Knudsen et al | 2017 | Norway | GBD estimates | GBD estimates |
| 44 | Kobyakova et al | 2014 | Tomsk Region of Russian | National Statistics (Territorial body of Federal service of state statistics) | NR |
| 45 | Koko et al | 2015 | Albania | Cross-sectional data | Cross-sectional data |
| 46 | Lai et al | 2004 | Estonia | Vital registration of Statistics Estonia | Estonian Health Insurance Fund database |
| 47 | Lai et al | 2006 | Estonia | Vital registration of Statistics Estonia | Estonian Health Insurance Fund database |
| 48 | Lai & Kohler | 2009 | Estonia | Vital registration of Statistics Estonia | Estonian Health Insurance Fund database |
| 49 | Lai et al | 2009 | Estonia | Vital registration of Statistics Estonia | Estonian Health Insurance Fund database |
| 50 | Lokkerbol et al | 2013 | Netherlands | NA | Netherlands Mental Health Survey and Incidence Study (NEMESIS) |
| 51 | Lapostolle et al | 2007 | France | Epidemiological Centre for Medical Causes of Death (Centre d’Epidémiologie sur les Causes Médicales de Décès) | WHO/GBD estimates |
| 52 | Lara et al | 2015 | Spain | GBD estimates | GBD estimates |
| 53 | Laursen et al | 2019 | Denmark | Danish Register of Causes of Death | NA |
| 54 | Lekhan & Kriachkova | 2019 | Ukraine | GBD estimates | GBD estimates |
| 55 | Lewis & Torgerson | 2017 | United Kingdom | Office for National Statistics (ONS; death registrations) | Empirical data (Delphi consensus estimates; 2014 Alzheimer's society estimates) |
| 56 | Ljung et al | 2005 | Sweden | National Cause of Death Registry | Population-based inpatient registers, national surveys, experts judgements |
| 57 | López-Jurado et al | 2008 | Spain | Vital Statistics from the Spanish National Statistics Institute | NA |
| 58 | Lunevicius et al | 2015 | Lithuania | WHO European Detailed Mortality Database, GBD, Statistics Lithuania, Lithuanian Cancer Registry, GLOBOCAN, WHO | WHO European Detailed Mortality Database, GBD, Statistics Lithuania, Lithuanian Cancer Registry, GLOBOCAN, WHO |
| 59 | Machado et al | 2011 | Portugal | GBD estimates | GBD estimates |
| 60 | Maertens de Noordhout et al | 2018 | Belgium | GBD estimates | GBD estimates |
| 61 | Mariotti et al | 2003 | Italy | ISTAT; Italian National Bureau of Census | NA |
| 62 | Mazzotti et al | 2019 | Netherlands | Netherlands Cancer Registry | NA |
| 63 | Melse & Kramers | 1998 | Netherlands | Dutch death registration | General practitioner registrations, national registries, and population surveys |
| 64 | Melse et al | 2000 | Netherlands | Dutch death registration | General practitioner registrations, national registries, and population surveys |
| 65 | Mesalles-Naranjo et al | 2018 | Scotland | National Records for Scotland (NRS); Office for National Statistics (ONS) | NA |
| 66 | Minicuci et al | 2011 | Italy | Cohort COPD data (other) | Cohort data [Hospital discharge records] |
| 67 | Monasta et al | 2019 | Italy | GBD estimates | GBD estimates |
| 68 | Moradi et al | 2006 | Sweden | National Board of Health and Welfare's cause of death register | WHO Statistical Information System |
| 69 | Murray et al | 2013 | United Kingdom | GBD estimates | GBD estimates |
| 70 | Nascimento et al | 2018 | Portugal | GBD estimates | GBD estimates |
| 71 | Newton et al | 2015 | England | GBD estimates | GBD estimates |
| 72 | Nielsen et al | 2004 | Greenland | Greenlandic Death Register | NA |
| 73 | Øverland et al | 2018 | Norway | GBD estimates | GBD estimates |
| 74 | Pereira et al | 2012 | Portugal | Official National Statistics | NA |
| 75 | Plana-Ripoll et al | 2019 | Denmark | Danish Register of Causes of Death | NA |
| 76 | Plass et al | 2014 | Germany | GBD estimates | GBD estimates |
| 77 | Rommel et al | 2018 | Germany | National Mortality Statistics | Claims data (WldO), Survey data (RK) |
| 78 | Sanchez-Valle et al | 2008 | Spain | California Developmental Disability System (adjust mort rates of CDDS to the Spanish population) | Literature; Western Australia Register for Autism Spectrum Disorders |
| 79 | Santric Milicevic  et al | 2009 | Serbia | Serbian Office of Statistics 'mortality database' (for Serbia) & GBD 2000 (for add analyses) | NA |
| 80 | Schopper et al | 2000 | Swiss canton | National mortality data base for the canton of Geneva | Published YLD/YLL data and/or primary GBD data (other) |
| 81 | Soriano et al | 2018 | Spain | GBD estimates | GBD estimates |
| 82 | Starodubov et al | 2018 | Russian Federation | GBD estimates | GBD estimates |
| 83 | Steel et al | 2018 | United Kingdom | GBD estimates | GBD estimates |
| 84 | Stockton et al | 2016 | Scotland | Scottish register of deaths | Routine administrative and survey datasets, largely patient-level records from health service activity |
| 85 | Tataru et al | 2019 | Romania | Arad Country Cancer Registry | GLOBOCAN 2013 data |
| 86 | Tollanes et al | 2018 | Norway | GBD estimates | GBD estimates |
| 87 | Tromme et al | 2016 | Belgium | Belgian Cancer Registry | Belgian Cancer Registry |
| 88 | Tyrovolas et al | 2018 | Greece | GBD estimates | GBD estimates |
| 89 | Ünüvar et al | 2006 | Turkey | Death statistics obtained from provincial and town centers in Turkey, hospital records and Directorate General of Security data | Population Census, Records obtained from government agencies, National surveys, national and international reports and articles |
| 90 | Valent & Zanier | 2015 | Italy | Regional mortality database of Friuli Venezia Giulia region | NA |
| 91 | Villaverde-Hueso et al | 2007 | Spain | National Statistics Institute | Literature |
| 92 | Vlajinac et al | 2006 | Serbia | Serbian Office of Statistics 'mortality database' | Cancer Registry of central Serbia for 1999, Cancer Regisrty of Vojvodina for 1998 |
| 93 | Vlajinac et al | 2006 | Serbia | Serbian Office of Statistics 'mortality database' | Registry of Myocardial Infraction and Stroke, Cindy and Monica Collaborative Center, Novi Sad |
| 94 | Vlajinac et al | 2008 | Serbia | Serbian Office of Statistics 'mortality database' | NA |
| 95 | Vondeling et al | 2018 | Netherlands | Netherlands Comprehensive Cancer Organization (IKNL); National Cancer Registries (NCR) | National Cancer Registries (NCR), Netherlands Comprehensive Cancer Organization (IKNL) |
| 96 | Witthaus et al | 1999 | Netherlands | Rotterdam study (literature; other) | Rotterdam study (literature; other) |
